# Hospital Branch of the Poor-Law Medical Service

**Published:** 1913-09-06

**Authors:** C. Thackray Parsons

**Affiliations:** Medical Superintendent of Fulham Infirmary.


### HOSPITAL BRANCH OF THE POOR-LAW MEDICAL SERVICE.

By C. THACKRAY PARSONS, M.D., Medical Superintendent of Fulham Infirmary.

To the newly qualified man, who wishes to extend his professional experience and knowledge before settling down in general practice or deciding upon one of the special branches of the profession, the indoor medical service of the Poor Law offers many advantages. But the chief posts in it are too few, their remuneration too often inadequate, promotion to them too often a matter of luck or influence, and too many Boards of Guardians exist with whom their chief medical officers must live in a constant and irritating state of warfare if they are to maintain and improve the efficiency of their infirmaries. Reforms which are bound to come sooner or later may change all this. Dealing with the service as it stands, however, it offers to the newly qualified man a number of paid appointments in which he can gain experience in a field more varied than that of the voluntary hospital, and in cases he seldom sees there, but which will form a great part of his work if he enters general practice. More than this, the conditions of the work are such that he will find himself thrown to a great extent upon his own resources, and compelled to develop his confidence in himself and his powers of initiative. At the same time he will find that in matters of doubt and difficulty there will always be accessible more experienced colleagues. Naturally, these advantages are not offered to the same degree by all the Poor-Law infirmaries. It will be better to avoid the smaller infirmaries and those which do not possess a resident medical superintendent and at least two assistant medical officers. The infirmaries of London and of the larger provincial towns, especially of those possessing universities or local medical schools, naturally offer the greatest advantages. Even here some discrimination is necessary. All the progressive infirmaries possess a dispenser and a medical superintendent's clerk, and those infirmaries where the medical officer is required to do the dispensing or to keep such books as the medical relief register should be carefully avoided. Other useful tests are the number of surgical operations performed annually and the possession of special apparatus. No infirmary should be selected in which the number of operations under general anæsthesia does not reach one hundred a year, or which does not possess an operating theatre, an $x$-ray apparatus, the provision necessary for bacteriological and pathological examinations, and the instruments for electrical treatment. Again, in some provincial infirmaries possessing resident medical superintendents the administrative separation of the infirmary from the workhouse is not complete. All such infirmaries should be studiously avoided.

The remuneration attached to the post of medical officer varies, and has shown a tendency to increase in recent years owing to the dearth of suitable candidates for these appointments. As a general rule, if the infirmary has two assistant medical officers, the junior will be paid £100 or £120 per annum, and the senior £150, rising to £175; where there are three assistants the salaries average £100 or £120 for the junior, £120 or £140 for the second, and £150, rising to £175 or £200, for the senior. In all cases the posts carry with them the usual residential allowances. No well-qualified man need accept indoor posts under the Poor Law with lower salaries than these.

### Infirmary and Hospital Posts Compared.

To most men, however, who seek for a post at an institution, for a year or two, after qualification, the money remuneration is a matter of secondary importance. The first question they will ask is, How does the experience to be gained at a Poor-Law infirmary compare with that to be gained at a voluntary hospital? To the man who means to devote himself to general surgery or to any of the usual special departments the Poor-Law infirmary cannot offer the same wealth of material or the same perfection of expensive apparatus for diagnosis and treatment which he will find at the large voluntary hospitals. On the other hand, the man who is going into private practice will find the experience and training to be gained whilst holding a post in a Poor-Law infirmary will supplement his hospital experience and will supply him with abundance of clinical material in just those diseases in which the voluntary hospital is weak. During his hospital career he will have had a more or less clear view of all the ordinary surgical operations, and will have performed some of them on the dead body; he will have given a small number of general anæsthetics under the direct supervision of an experienced anæsthetist; he will have seen the whole course of all the ordinary acute diseases which need rest in bed; and he will have attended clinical demonstrations on cases of insanity and the acute infectious fevers. But all the time he will necessarily be acting under close supervision; he will not have been personally responsible for the ordering of any treatment, his examinations of patients will be checked and directed by the visiting staff, he will probably never have followed the course of such diseases as chronic joint and nervous complaints, etc., except under the unsatisfactory conditions of the out-patient department. It is quite possible for a man to become qualified without ever having acted as the chief assistant to the surgeon at an important operation, without ever having watched the course of a case of measles or whooping-cough, and without ever having seen the treatment of a bedridden patient crippled with gout or rheumatoid arthritis, or helpless from advanced tabes dorsalis, or other chronic nervous affection. It is the lack of experience of these and similar diseases, and the fact that the student is never required to initiate treatment or to act upon his own responsibility that are the greatest drawbacks to the present system of medical education; and it was the strength of the old apprentice and unqualified-assistant systems that this lack was supplied by them. It is also just in these directions that the Poor-Law infirmary is able to supplement the hospital training.

Assistant Medical Officers' Opportunities.

In most Poor-Law infirmaries the assistant medical officer will have charge of five or six wards, with 120 to 150 patients. On his full duty days he will usually have to examine, allocate to their proper wards, and initiate the treatment of all new admissions. The medical superintendent, as a matter of routine, sees all the new admissions also, and keeps in touch with the progress of the more serious or interesting cases, and naturally often suggests or alters a line of treatment. Nevertheless the assistant medical officer is directly responsible for the treatment of most of the cases in his wards, and for the detection of new developments and complications in the course of the disease. At the same time he is required to bring to the notice of the medical superintendent all cases of doubt or difficulty. He is expected to make any special investigation necessary himself, to use any special instruments of diagnosis such as the ophthalmoscope, laryngoscope, cystoscope, sigmoidoscope, sphygmomanometer, etc., to examine his patient with the $x$-rays, to make a blood count, to make a bacteriological examination of sputum or other material, to obtain and make cytological examinations of cerebro-spinal fluid or of pathological exudates, and so on. If his hospital training has left him unable to carry out any such investigation, he soon picks up the necessary technique from his senior colleagues, who are always at hand to help him and check his results. The important point is that he is expected to acquire the necessary dexterity if he does not already possess it, and is not expected to delegate the work to someone else. In this way he obtains a practical and all-round acquaintance with methods of diagnosis which in the hospitals are usually carried out in the special departments, and in which the ordinary medical student is therefore often only imperfectly practised. At the same time, the fact that he is compelled to rely upon himself for the ordinary routine work of the wards gives him self confidence, trains him in the practical application of his book knowledge of treatment, and renders him tactful and self-reliant in dealing with patients, whilst the amount of work he has to get through makes him quick in his methods. Moreover, he finds he has in his wards many cases of acute medical and surgical diseases with which his hospital experience has rendered him familiar; but he finds also half his beds filled with cases with which his practical experience hitherto has been very limited. Rheumatoid arthritis and chronic gout in all stages will test all his powers in his attempts to cope with the pain and to prevent or delay the progressive involvement and fixation of the joints. Hemiplegias and chronic spinal and nerve degenerations will provide him with a fine field for diagnosis and for observation in the results of treatment by exercises, massage, and electricity. He will find chronic bronchitis and emphysema and the nervous, arterial, glandular, and other degenerations of old age present difficulties of prognosis and of treatment that he has probably never realised. He will be able to study from day to day, and week to week, the terrible closing scenes of carcinoma of the mouth, the breast, the uterus, and rectum, and will learn something of what can be done by surgical and medical methods to alleviate the suffering, and how often all remedial efforts fail to produce complete comfort and relief of pain. He will discover that ulcered legs are frequently far more difficult to cure than his text-book reading led him to surmise. He will be able to watch, perhaps for the first time, the process of cure of a case of scabies, and to appreciate the difficulties of efficiently treating cases of chronic eczema and other skin complaints. Cases of epilepsy, of chronic and advanced phthisis, of gonorrhœa and syphilis, which he has only seen at intervals in the out-patient department, he will now have continuously under his eye, and will be able to follow their progress and their reaction to treatment. Cases of minor ailments the Poor-Law infirmary must admit, because the conditions of the home preclude satisfactory treatment and the hospitals have no room. These cases, which constitute a large part of the work of the private practitioner, afford to the infirmary medical officer experience which will be invaluable. Quite as important are the infectious diseases which are shut out from the general hospitals—measles, erysipelas, whooping-cough, chicken-pox, rötheln, and mumps. No institutions except some of the fever hospitals and the Poor-Law infirmaries admit these cases to their wards. To the maternity wards cases of difficulty are often sent in for delivery, so that the use of forceps, turning, cephalotripsy, abdominal and vaginal Cæsarian section, and other obstetric manœuvres are not infrequently required. Again, cases of puerperal fever and of ophthalmia neonatorum occurring amongst the poor are practically always sent into the local Poor-Law infirmary.

On the surgical side the opportunities are quite as valuable. In most infirmaries each medical officer can count upon being required to give from fifty to 100 anæsthetics each year, excluding the administration of nitrous oxide for short operations.

Another field in which the infirmary offers valuable experience not to be obtained in the general hospital is to be found in the insane wards. In these, cases of acute insanity are received pending their recovery or their removal to the asylum, and cases of chronic and harmless insanity are often kept under permanent detention orders. Here the infirmary medical officer sees all forms of insanity.

The value of the experience at the large Poor-Law infirmaries is recognised by the University of London, which accepts a certificate of a six months' appointment at one of them in lieu of the certificate of five years in private practice required of candidates for the M.D.

